# Inflammatory activation and immune cell infiltration are main biological characteristics of SARS-CoV-2 infected myocardium

**DOI:** 10.1080/21655979.2021.2014621

**Published:** 2022-01-17

**Authors:** Rui Zhang, Xi Chen, Wenjie Zuo, Zhenjun Ji, Yangyang Qu, Yamin Su, Mingming Yang, Pengfei Zuo, Genshan Ma, Yongjun Li

**Affiliations:** Department of Cardiology, Zhongda Hospital, School of Medicine, Southeast University, Nanjing, P.R. China

**Keywords:** SARS-CoV-2, cardiomyocyte, heart injury, COVID-19, bioinformatics

## Abstract

The severe acute respiratory syndrome coronavirus-2 (SARS-CoV-2) can target cardiomyocytes (CMs) to directly invade the heart resulting in high mortality. This study aims to explore the biological characteristics of SARS-CoV-2 infected myocardium based on omics by collecting transcriptome data and analyzing them with a series of bioinformatics tools. Totally, 86 differentially expressed genes (DEGs) were discovered in SARS-CoV-2 infected CMs, and 15 miRNAs were discovered to target 60 genes. Functional enrichment analysis indicated that these DEGs were mainly enriched in the inflammatory signaling pathway. After the protein-protein interaction (PPI) network was constructed, several genes including CCL2 and CXCL8 were regarded as the hub genes. SRC inhibitor saracatinib was predicted to potentially act against the cardiac dysfunction induced by SARS-CoV-2. Among the 86 DEGs, 28 were validated to be dysregulated in SARS-CoV-2 infected hearts. Gene Set Enrichment Analysis (GSEA) analysis of Kyoto Encyclopedia of Genes and Genomes (KEGG) showed that malaria, IL-17 signaling pathway, and complement and coagulation cascades were significantly enriched. Immune infiltration analysis indicated that ‘naive B cells’ was significantly increased in the SARS-CoV-2 infected heart. The above results may help to improve the prognosis of patients with COVID-19.

## Introduction

Coronavirus disease 2019 (COVID-19), which results from the severe acute respiratory syndrome coronavirus 2 (SARS-CoV-2), is spreading all over the world and thus causing a serious global health crisis [1]. SARS‐CoV‐2 has a high affinity to angiotensin‐converting enzyme 2 (ACE2) and ACE2 plays a pivotal role in the pathogenesis of this infectious disease. ACE2 is ubiquitous and widely expressed in human tissues especially in alveolar cells [2]. As expected, the lung is one of the most important target organs during the SARS-CoV-2 attack on the body. It has been reported that some patients progress to severe respiratory failure and acute respiratory distress syndrome (ARDS) which requires mechanical ventilation, and that the mortality rate of these patients is as high as 20–40% [3].

Except for the lung, ACE2 is also highly expressed in the cardiovascular system [4]. In fact, in the systemic symptoms caused by SARS-CoV-2 infection, myocardial injury is one of the important pathogenic features which further increases the risk of mortality [5]. According to previous reports on SARS-CoV, the existence of viral RNA and macrophage infiltration could be simultaneously detected in clinical heart samples, suggesting direct and indirect viral-induced damage to the myocardium [6]. Perez-Bermejo JA et al [7] performed experiments and discovered that SARS-CoV-2 was more inclined to infect cardiomyocytes (CMs) over fibroblasts and endothelial cells. However, the downstream effects on cardiomyocytes after infection with this virus are not delineated.

Therefore, in this study, the differentially expressed genes (DEGs) in SARS-CoV-2 infected three types of human CMs were re-analyzed, and the overlapping genes were enrolled for analysis of protein-protein interaction (PPI), Gene Ontology (GO), Kyoto Encyclopedia of Genes and Genomes (KEGG), as well as their upstream regulators. Besides, the potential therapeutic drugs are predicted. Furthermore, these DEGs in SARS-CoV-2 infected human hearts were re-analyzed and used to validate the DEGs of in-vitro results. Finally, based on the in vivo results, Gene Set Enrichment Analysis (GSEA) analysis was performed and immune infiltration was evaluated. We hope that the above bioinformatics analysis will show the biological characteristics during SARS-CoV-2 infected myocardium.

## Methods

### Bioinformatics datasets

Three datasets GSE150392, GSE151879 and GSE169241 were obtained from Gene Expression Omnibus (GEO, https://www.ncbi.nlm.nih.gov/geo/) database. In GSE150392, human induced pluripotent stem cell-derived cardiomyocytes (hiPSC-CMs) were infected with Mock or SARS-CoV-2. In GSE151879, human embryonic stem cells-derived cardiomyocytes (hESC-CMs) and adult human cardiomyocytes (AHCMs) were both infected with Mock or SARS-CoV-2. In GSE169241, heart samples of three COVID-19 patients and five controls were collected. All the gene expression profiles of the aforementioned datasets were detected by high-throughput sequencing.

### DEGs analysis

The raw-count of GSE150392 [8], GSE151879 [9], and GSE169241 [10] were enrolled for detecting DEGs between SARS-CoV-2 infected and uninfected myocardium. Networkanalyst (https://www.networkanalyst.ca/) webtool, a collection of online user-friendly bioinformatic tools [11], was used to analyze the above DEGs after normalizing the data using DESeq2 [12]. The criteria for DEGs are as follows: adjusted P-value < 0.05, and log_2_ fold change (FC) ≥ 1 or ≤ −1.

### Identification of upstream TFs and miRNAs

The expression of DEGs could be specifically regulated by TFs at transcriptional level [13] and miRNAs at post-transcriptionally [14]. The transcription factors (TFs) of these DEGs were predicted with FunRich (http://www.funrich.org/) software [15], and the top 10 TFs were picked out. The upstream miRNAs were searched on miRNET website (https://www.mirnet.ca/) [16], and the miRNA-mRNA network was subsequently constructed using the website.

### Functional enrichment analysis

GSEA is a knowledge-based approach for analyzing groups of genes that share common biological functions, chromosomal location, or regulation [17]. GO analysis mainly reflects three independent ontologies of group genes: molecular function (MF), biological process (BP) and cellular component (CC) [18]. WEB-based Gene SeT AnaLysis Toolkit (WebGestalt, http://www.webgestalt.org/), a user-friendly functional enrichment analysis webtool [19], was applied for GSEA and GO analysis. KEGG database provided biological pathway information of DEGs enrichment [20]. Enrichr website (https://amp.pharm.mssm.edu/Enrichr/), a comprehensive, freely available gene set enrichment analysis web server [21], was used to explore pathway information.

### PPI network construction and analysis

The ‘Search Tool for Retrieval of Interacting Genes/Proteins’ database (STRING, https://string-db.org/) integrates all available information regarding protein-protein interaction [22], and all DEGs were mapped into the database to construct the complete PPI network. Subsequently, the network was imported into Cytoscape software (https://cytoscape.org/), an open-source software platform for visualizing complex networks [23], to screen out hub nodes with plug-in ‘cytoHubba’ according to the indexes Degree, Betweenness, and Closeness. Furthermore, the plug-in ‘MCODE’ was used to extract the clustering function modules. The criteria used was as follows: degree cutoff = 2, node density cutoff = 0.1, node score cutoff = 0.2, *k*-core = 2 and max depth = 100.

### Potential therapeutic drugs prediction

The Connectivity Map (cMap) source can provide information of connection between gene expression signatures and small molecules, genes or disease [24]. In order to search for drugs against virus-induced myocardial injury, drug repurposing analysis was performed on the cMap database by using the online application CLUE (https://clue.io) [25]. The first 10 molecules with the lowest scores were collected.

### Immune infiltration analysis

CIBERSORT (https://cibersort.stanford.edu/) is an analytical online webtool that provides an estimation of the abundance of member cell types in a mixed cell population by using gene expression data [26]. The immune infiltration of 22 kinds of immunocytes was evaluated with CIBERSORT and analyzed with the wilcoxon test method to compare the difference between infected and uninfected hearts. A p-value < 0.05 was considered statistically significant. Pearson correlation between infiltrating immunocytes was calculated in GraphPad Prism 8.0.

## Results

In the present research, we aimed to explore the biological characteristics of SARS-CoV-2 infected myocardium by bioinformatic tools. After re-analyzing the datasets of SARS-CoV-2 infected CMs, the DEGs were picked out and enrolled for GO, KEGG analysis, and PPI network construction. The upstream regulator including TFs and miRNAs that are potentially responsible for the expression of DEGs were screened. In order to find possible therapeutic drugs, the DEGs were collected for analysis in the specific database. Finally, the dataset of the hearts of COVID-19 patients was re-analyzed to confirm the in-vitro results, and further used for detecting the immune infiltration.

### Identification of DEGs during SARS-CoV-2 infected cardiomyocytes

**Figure 1.**
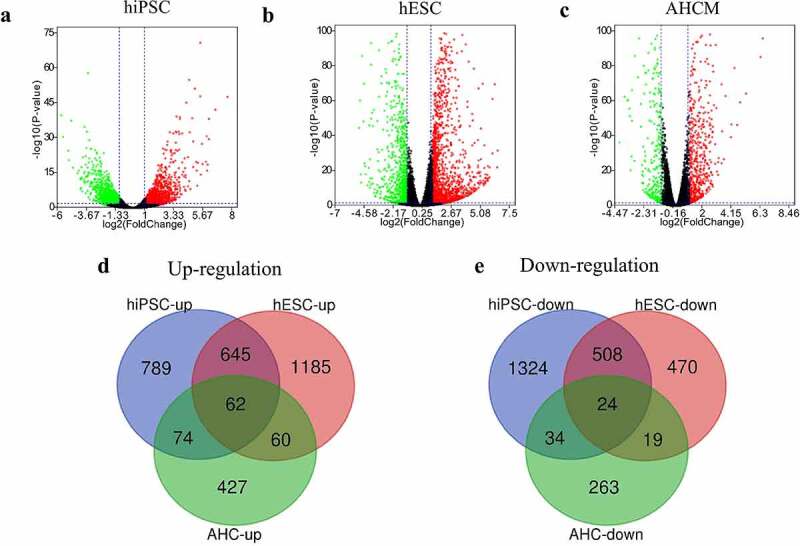
Analyze the DEGs between mock and SARS-CoV-2 infected CMs. Volcano plot showed DEGs as red dot represent up-regulated and green dot represent down-regulated genes in hiPSC-CMs (a), hESC-CMs (b), and AHCMs (c) respectively. (d) The venn diagram indicated 62 genes were upregulated in all three datasets. (e) The venn diagram indicated 24 genes were downregulated in all three datasets.

In GSE150392, 1570 genes were found to be upregulated while 1890 genes were downregulated in SARS-CoV-2 infected hiPSC-CMs (Figure 1(a)). In GSE151879, there were 1952 upregulated and 1021 downregulated genes in SARS-CoV-2 infected hESC-CMs (Figure 1(b)), and 623 upregulated and 340 downregulated genes in SARS-CoV-2 infected AHCMs (Figure 1(c)).

In order to more accurately screen out the DEGs after the virus interfered with CMs, we took the intersection of up- and down-regulated DEGs among three groups. The results showed that there were 62 genes up-regulated in all three groups (Figure 1(d), Table 1), and only 24 genes were down-regulated in all three groups (Figure 1(e), Table 1). In short, these 86 DEGs were considered to be the genes that are most likely differentially expressed after SARS-CoV-2 infected the CMs.

**Table 1.** The overlapping DEGs in datasets of SARS-CoV-2 infected CMs

DEGs	Genes
Up-regulated	TNIP3, SLC38A5, CD38, TNFRSF9, RIPK4, STARD13, NFKB2, BDKRB1, PCK2, PDE4C, LIF, IL27RA, BMP6, NUAK1, NEDD4L, DSE, NFKBIZ, PTGS2, WNK4, IL4I1, CDKN2B, PIK3CD, TNFAIP3, ZSWIM4, PTGER4, SLC22A3, CD14, PCED1B, EBI3, GATA3, IRAK2, POSTN, SERTAD4, SUSD3, SLC2A6, NFE2L3, KLF5, PID1, RUNX2, LOX, IL32, RPS6KA1, SKIL, NFKBIA, PFKFB4, NOD2, BHLHE41, AMIGO2, TYMP, RFLNA, IRF1, ELF3, PTPRB, PAPPA, RUNX1, CXCL8, LTBP2, ZC3H12A, FRMD6, ETV4, CPM, DRAM1, FLNB, TNFSF18, SRGN, CCL2, IL6, SYT1, RORA, INHBA, SERPINE2, ARRDC3, PRR15, SPINT1, SHROOM3, SERPINE1, TNFAIP2, NEDD9, IL11, KLF10, BCAT1, TFPI2, ICAM5, BIRC3
Down-regulated	TACC2, KLHL41, NEBL, SELENOP, CSRP3, MLIP, CAMK2B, LRRC2, MYOZ2, HAND2, SNTA1, PLCXD3, SYNPO2L, PPFIA2, LBH, DRD1, PLN, HSPB3, COL21A1, ADCY5, VSNL1, STRIP2, RCAN2, OXCT1, FABP3, INKA2, MAN1C1, MYH11, IL17RB, BDH1, SREBF2-AS1, GJA3, PPM1L, GPX3

### Prediction of upstream TFs and miRNAs

**Figure 2.**
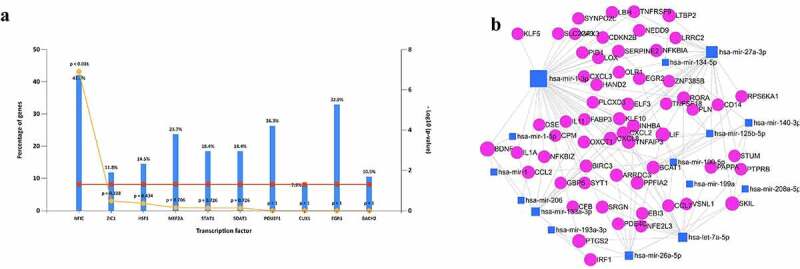
Predict the upstream regulators of the overlapped 86 DEGs. (a) The potential upstream TFs including NFIC, ZIC1. (b) Predict upstream miRNAs and construct the miRNA-mRNA network in muscle tissue. Blue quadrilateral was miRNA and red circular was DEGs.

There were several TFs predicted to regulate these 86 DEGs. As shown (Figure 2(a)), only NFIC was significantly enriched, and nine others including ZIC1, HSF1, MEF2A, STAT1, SOAT1, POU2F1, CUX1, EGR1 and BACH2 showed no significance.

Besides, the upstream miRNAs were predicted and 15 miRNAs were found to potentially regulate the expression of 60 of 86 DEGs, and they formed a complex network containing 132 edges (Figure 2(b), Supplementary Table 1). Among all the miRNAs, miR-1-3p and miR-27a-3p had the most target genes.

### Analyze GO and KEGG

**Figure 3.**
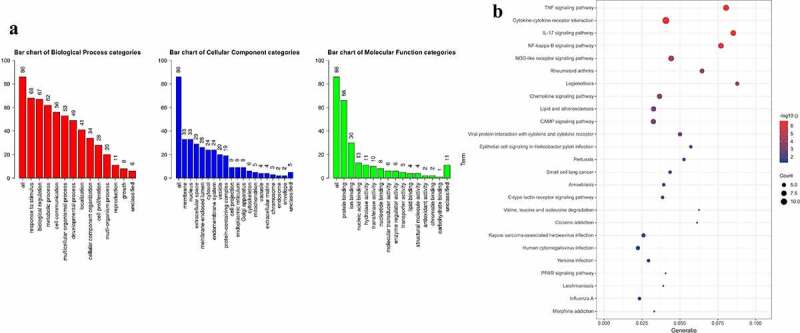
Analyze the GO and KEGG pathway enrichment of 86 DEGs. (a) Bar chart showed results of Biological process (red), Cellular component (blue), and Molecular function (green). (b) Bubble diagram showed the KEGG pathways enrichment results. The size of the dot represents count, and color represents -log_10_ adjusted p value.

We further assessed the biological role of 86 DEGs with GO analysis. The results showed that these DEGs were enriched in response to stimulus, biological regulation, and metabolic process under BP categories (Figure 3(a)). In terms of MF, the functions of DEGs were mainly involved in protein binding, ion binding and nucleic acid binding (Figure 3(a)). These DEGs were distributed in different structures of cells including membrane, nucleus, and extracellular space (Figure 3(a)).

Biological pathway was analyzed with KEGG database [20]. As shown (Figure 3(b)), several signaling pathways were significantly enriched including TNF signaling pathway, Cytokine-cytokine receptor interaction, IL-17 signaling pathway, NF-kappa B signaling pathway, etc., indicating that the function of DEGs was closely related to inflammatory phenotype.

### Construction of the PPI network and hub genes analysis

**Figure 4.**
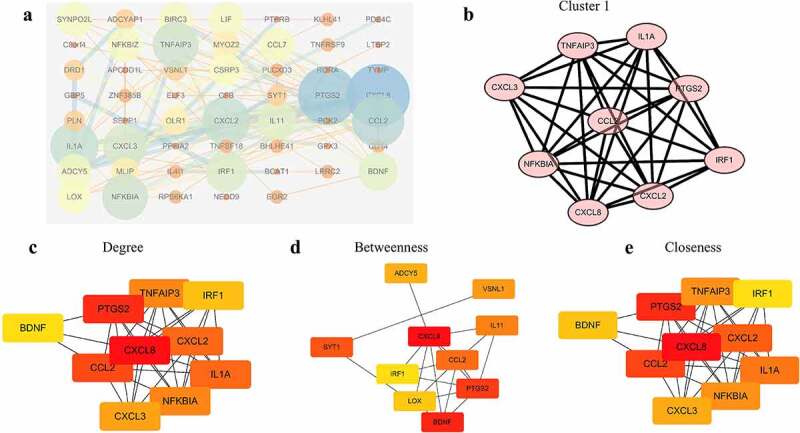
Construct the PPI network and analyze the properties of nodes. (a) The whole PPI network containing 54 nodes and 121 edges. (b) The significant module with score 8.75. (c) The top 10 genes according to the parameter degree. (d) The top 10 genes according to the parameter betweenness. (e) The top 10 genes according to the parameter closeness.

To construct the PPI network, we mapped all the 86 DEGs into STRING database and generated a complex network structure, in which 54 nodes and 121 edges were retained (Figure 4(a)). After ‘MCODE’ [27] analysis, we revealed two modules where the first one scored 8.75 (Figure 4(b)). ‘cytoHubba’ [28] was another way to evaluate the nodes through ‘degree’, ‘betweenness’ and ‘closeness’. The top 10 genes under three dimensions were displayed (Figure 4(c-e)). Altogether, several genes such as CCL2, CXCL3, CXCL8, CXCL2, IRF1, etc. were potential hub ones.

### Predicting potential anti-SARS-CoV-2-induced cardiomyocyte disorder drugs

In order to find a way to repair the damage, we further explored the possible drugs based on the 86 dysregulated genes. There was a total of 2429 compounds enrolled and scored (Supplementary Table 2), and the ten most likely therapeutic drugs were PIK-75, ZG-10, triptolide, peucedanin, PD-0325901, phenylbutyrate, NSC-23766, saracatinib, PD-98059, and XMD-892, all of which with scores less than −90 (Table 2).

**Table 2.** Potential drugs for the treatment of SARS-CoV-2 induced CMs dysfunction

Rank	Score	Name	Description
1	−98.03	PIK-75	DNA protein kinase inhibitor
2	−97.34	ZG-10	JNK inhibitor
3	−97.04	triptolide	RNA polymerase inhibitor
4	−95.93	peucedanin	Apoptosis stimulant
5	−95.86	PD-0325901	MEK inhibitor
6	−95.09	phenylbutyrate	HDAC inhibitor
7	−93.67	NSC-23766	Ras GTPase inhibitor
8	−93.02	saracatinib	SRC inhibitor
9	−92.9	PD-98059	MEK inhibitor
10	−92.53	XMD-892	MAP kinase inhibitor

### Validation of the DEGs expression in the dataset of SARS-CoV-2 infected heart

**Figure 5.**
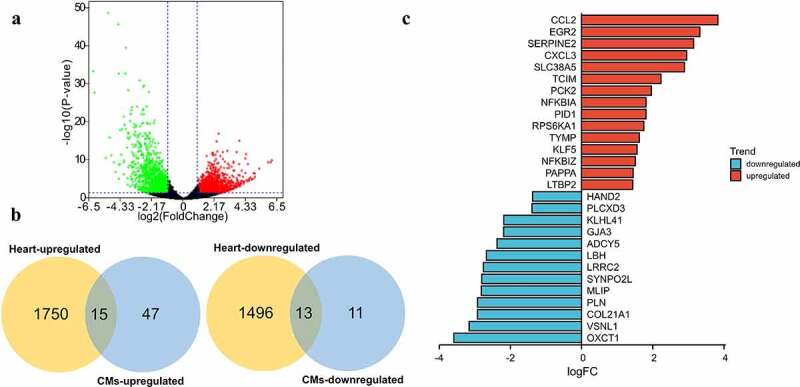
Validate the expression of the 86 DEGs in dataset of COVID-19 heart samples. (a) Volcano plot showed DEGs as red dot represent up-regulated and green dot represent down-regulated genes in COVID-19 heart. (b) The overlapping of up-regulated DEGs and downregulated DEGs between SARS-CoV-2 infected CMs and heart. There were 15 of 62 overlapped DEGs upregulated and 13 of 24 overlapped DEGs downregulated in SARS-CoV-2 infected heart. (c) The logFC value of above 28 DEGs (red marked 15 upregulated and blue marked 13 downregulated) in SARS-CoV-2 infected heart.

To fully understand the biological characteristics of virus-infected myocardium, we re-analyzed the DEGs between COVID-19 infected hearts and uninfected hearts. As indicated (Figure 5(a)), there were 1765 upregulated and 1509 downregulated DEGs in the SARS-CoV-2 infected heart. More than that, 15 upregulated and 13 downregulated genes of the 86 DEGs were also dysregulated in heart samples (Figure 5(b)), showing that the in vitro SARS-CoV-2 infected CMs could simulate in vivo infection in the heart to some extent. All the 28 DEGs were ordered according to the size of their logFC in GSE169241 (Figure 5(c)), and CCL2 was found to be the most increased while OXCT1 decreased the most in SARS-CoV-2 infected hearts.

**Figure 6.**
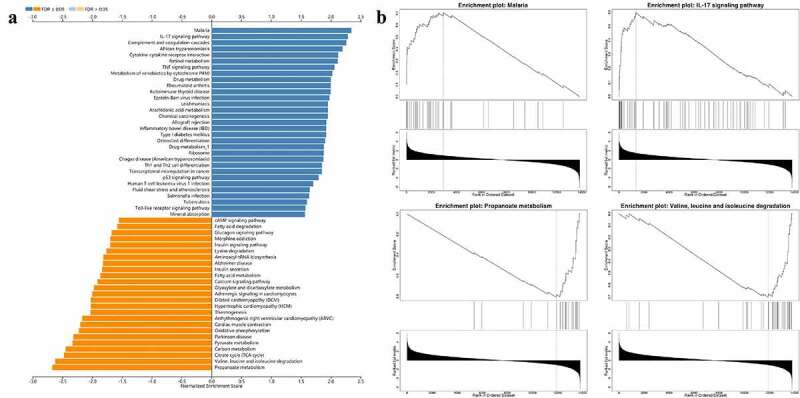
Biological characteristics of SARS-CoV-2 infected heart. (a) GSEA analysis indicated KEGG results of infected heart in COVID-19 patients. There were totally 56 items listed. (b) Example items of the positive and negative enrichment set including ‘Malaria’, ‘IL-17 signaling pathway’, ‘Propanoate metabolism’, and ‘Valine, leucine and isoleucine degradation’.

We also performed GSEA analysis to uncover the biological pathways involved. As shown, 31 KEGG terms including ‘Malaria’, ‘IL-17 signaling pathway’, ‘Complement and coagulation cascades’, etc. were positively associated with the pathological state, while 25 terms including ‘propanoate metabolism’, ‘valine, leucine, and isoleucine degradation’, and ‘Citrate cycle (TCA cycle)’, etc. were negatively associated with the disease progression (Figure 6(a,b)).

### Assessing the immune infiltration in the SARS-CoV-2 infected heart

**Figure 7.**
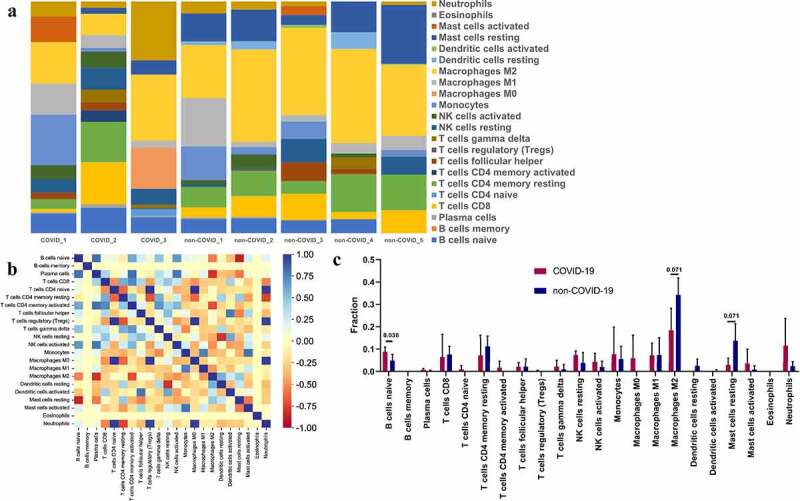
Immune infiltration in SARS-CoV-2 infected heart. (a) Barplot showed the composition of immunocytes in SARS-CoV-2 infected hearts and uninfected hearts. A total of 22 immune cells were included. (b) Heatmap showed the Pearson correlation between 22 kinds of immunocytes. The blue represented positive correlation, red represented negative correlation and yellow represented no correlation between two cells. (c) The content of 22 types of immunocytes in hearts of COVID-19 and none-COVID-19 patients were compared. Propotion of ‘B cells naïve’ was significantly higher in COVID-19 hearts than in control hearts.

The infection of SARS-CoV-2 was able to induce abnormal response of the inflammatory signal pathway. We, therefore, assessed the immune infiltration of SARS-CoV-2 infected hearts (Figure 7(a)). The results showed that there were closely positive correlations and negative correlations between the infiltration fraction of multiple inflammatory cells (Figure 7(b)). Furthermore, the proportion of ‘naïve B cells’ was significantly upregulated (P = 0.036) after the SARS-CoV-2 infection (Figure 7(c)), while ‘Macrophages M2ʹ and ‘Mast cells resting’ showed a downward trend (P = 0.071 for both). This provides a clue that naive B cells may be the focus of intervention.

## Discussion

There is no doubt that SARS-CoV-2 induced COVID-19 mainly focused on the respiratory system and results in severe respiratory failure ultimately causing death [29]. Besides, one more important reason for the death of COVID-19 patients is the destruction of the cardiovascular system. Myocardial injury is common during SARS-CoV-2 infection, and researchers have found that it is significantly associated with a higher risk of mortality [30,31]. Therefore, it is urgent to explore the biological effects of SARS-CoV-2 on the heart.

Genes are key participants in life activities and disease evolution. In this study, we firstly collected three independent datasets from the GEO database and re-analyzed them. These three datasets used SARS-CoV-2 or mock treatment to interrogate a response from cardiomyocytes originating from hiPSC, HESC, and adult humans, and detected via high throughput sequencing. The final results revealed thousands of differentially expressed genes in each dataset. However, only 86 DEGs were concurrently up-regulated or down-regulated in all datasets, indicating the existence of several internal biological differences among the above CMs from the three sources when exposed to SARS-CoV-2. Therefore, these overlapped genes were selected as the most potential abnormally expressed factors in SARS-CoV-2 infected CMs. To verify the reliability of these genes, we re-analyzed the dataset of heart samples from clinical COVID-19 patients and uninfected individuals and found that 28 genes of the 86 DEGs were also significantly differentially expressed in vivo, suggesting that the in vitro model was reliable to some extent.

To construct the integrated regulatory network, we predicted the upstream transcription factors and miRNAs with bioinformatic tools. In terms of the transcription factors listed, only NFIC was significantly enriched while others showed no significance. MiRNAs are tissue-specific regulators that can inhibit gene expression. We further predicted the possible miRNAs targeting the 86 DEGs in muscle to construct a precise network. We found 15 miRNAs that potentially regulate a total of 60 genes from 86 DEGs and miR-1-3p is most widely associated with these DEGs. According to previous research, miR-1-3p was increased in peripheral blood and positively correlated with the level of myocardial damage in acute viral myocarditis [32] and therefore whether its circulating form could contribute to diagnose and even judge the prognosis in SARS-CoV-2 induced myocardial injury is worth exploring. Besides, its extensive association with multiple DEGs suggests that it is also a potentially valuable intervention target.

There is still insufficient understanding of this virus-induced myocardial disorder. Based on all dysregulated genes, we evaluated the characteristics of the above biological dysfunction through GO and KEGG analysis. In terms of the biological process in GO, we found that response to stimulus, biological regulation, and metabolic process were significantly enriched. The analysis of biological pathways by KEGG provides abundant information. As indicated in the analysis, these DEGs were enriched in ‘TNF signaling pathway’, ‘Cytokine-cytokine receptor interaction’, ‘IL-17 signaling pathway’, and ‘NF-kappa B signaling pathway’, whose roles were also vital in other viral myocarditis [33,34]. This suggested that anti-inflammatory strategies will likely help to combat myocardial injury in COVID-19 patients. We also focused on the KEGG signaling pathway by GSEA analysis with the results of in vivo dataset, and confirmed the importance of ‘IL-17 signaling pathway’ in the disease progression once again. In fact, activation of IL-17 signaling pathway, to a certain extent, is a landmark event of SARS-CoV-2 infection [35], and mediated the cytokine storm caused by SARS-CoV-2 infection [36]. More than that, ‘Complement and coagulation cascades’ were proven to actively participate in this cardiac anomaly, which highly suggests that myocardial injury in COVID-19 may be partly from microcirculatory thrombosis.

To identify the key genes responsible for exacerbating SRARS-CoV-2 damage to CMs, we constructed a PPI network with all 86 DEGs and further analyzed the nodes by using ‘MCODE’ and ‘cytohubba’ in cytoscape software. Several genes including CCL2, CXCL8, PTGS2, and IRF1 were considered to be hub genes because they were screened out according to different standards simultaneously. Chemokine (C-C-motif) ligand 2 (CCL2), also known as monocyte chemoattractant protein-1 (MCP-1), is a member of the chemokine family, and responsible for attracting leukocytes to sites of infection or injury to mediate defense and repair [37]. In a recent research, SARS-CoV-2 infected CMs could induce CCL2 secretion and further recruit monocyte[9][], indicated a vital role of CCL2 in the pathological mechanism. CXCL8, also called IL-8, is a prototypical member of the CXC family, and is usually responsible for the recruitment and activation of inflammatory cells through interaction with cell surface receptor GPCR, CXCR1 and CXCR2 [38]. In COVID-19, CXCL8 expression was higher only in severe patients, and positively correlated with the percentage of neutrophils [39], which proposed that CXCL8 leads to deterioration of the condition by recruiting neutrophils. CXCL8, along with IL-37 and CRP, could be combined into a highly sensitive model to effectively differentiate severe cases from moderate ones in the COVID-19 population [40]. Therefore, the above hub genes such as CCL2 and CXCL8 may be key participants and diagnostic indicators in COVID-19 patients who experienced myocardial injury.

Due to the fact that these hub genes were closely associated with inflammatory cells in function, we also evaluated the immune infiltration in the injured heart sample with the in vivo dataset. After the execution in the CIBERSORT webtool and subsequent statistical analysis, only the proportion of ‘naive B cells’ was significantly increased in SARS-CoV-2 infected hearts, while ‘Macrophages M2ʹ and ‘Mast cells resting’ decreased with parameter p-value close to 0.05. The naive B lymphocytes are a major mediator of adaptive immunity in mammals [41]. In a recent research by Wu et al [42]. the immune infiltration landscape in the lung tissues of COVID-19 patients was estimated, and naive B cells infiltration was found to be high and may be the main cause of the over-active humoral immune response. Furthermore, it was found that the increased B cell infiltration was associated with poorer clinical outcomes, and may act as a trigger of severe respiratory and pulmonary symptoms of COVID-19. Several specific molecules that can target B cells have been shown to be beneficial against the COVID-19 pandemic [43,44]. Therefore, the increased B cell infiltration is a potential culprit of functional deterioration in the hearts of COVID-19, and appropriate inhibition of naive B cells might be an effective strategy to alleviate COVID-19-induced heart dysfunction/ damage.

Exploring therapeutic drugs is of great significance to improve the prognosis of COVID-19 patients. We matched 86 DEGs into the cMAP database and obtained scores of all 2429 small molecules. A series of compounds were identified to potentially reduce the SARS-CoV-2 induced CMs injury. The antiviral saracatinib, one of the most likely therapeutic drugs, has been considered to be effective against SARS-CoV-2 in two different research models [45,46]. However, whether saracatinib or other predicted small molecules could prevent myocardial tissue damage caused by SARS-CoV-2 remains to be confirmed.

## Conclusion

In conclusion, our study identified potential biomarkers in SARS-CoV-2 infected myocardium, and found potential immune cell intervention targets and valuable therapeutic drugs. However, further studies are needed to confirm our preliminary results.

## Supplementary Material

Supplemental Material

## Data Availability

The datasets analyzed was acquired from GEO (http://www.ncbi.nlm.nih.gov/geo).

## Highlights

- The miRNA-mRNA network and PPI network will improve our understanding of the disease development and can be applied for exploring potential disease biomarkers.
- Fraction of ‘naive B cells’ was significantly increased in SARS-CoV-2 infected myocardium, and may be a potential intervention target.
- Several small moleculars especially saracatinib were believed to fight disease progression.
